# Coping, Resilience, and Perceived Stress in Individuals with Internet Gaming Disorder in Taiwan

**DOI:** 10.3390/ijerph18041771

**Published:** 2021-02-11

**Authors:** Pai-Cheng Lin, Ju-Yu Yen, Huang-Chi Lin, Wei-Po Chou, Tai-Ling Liu, Chih-Hung Ko

**Affiliations:** 1 Department of Psychiatry, Kaohsiung Municipal Ta-Tung Hospital, Kaohsiung Medical University, Kaohsiung 801, Taiwan; superfoxcat@yahoo.com.tw (P.-C.L.); yenjuyu@cc.kmu.edu.tw (J.-Y.Y.); 2 Department of Psychiatry, Faculty of Medicine, College of Medicine, Kaohsiung Medical University, Kaohsiung City 807, Taiwan; cochigi@yahoo.com.tw (H.-C.L.); dai32155@gmail.com (T.-L.L.); 3 Department of Psychiatry, Kaohsiung Medical University Hospital, Kaohsiung Medical University, Kaohsiung City 807, Taiwan; webber1007@gmail.com; 4 Department of Psychiatry, Kaohsiung Municipal Siaogang Hospital, Kaohsiung Medical University, Kaohsiung City 812, Taiwan

**Keywords:** internet gaming disorder, coping, stress, resilience

## Abstract

Aim: Gaming escapism is an essential factor for developing internet gaming disorder (IGD). We evaluated coping strategies, resilience, stress, and depression in individuals with IGD. Methods: We included 69 participants with IGD and 138 controls (69 regular gamers and other non-gamers) in Taiwan. The self-reported coping strategies, coping stress with gaming, resilience, perceived stress, and depression were assessed. Results: Participants with IGD had higher dysfunctional coping, coping stress by gaming, perceived stress, and depression, as well as lower problem-focused coping and resilience. Regression analysis revealed that coping by gaming was associated with dysfunctional coping mechanisms, particularly venting and self-distraction. Compared with participants with adequate resilience, those with lower resilience had higher perceived stress, depression, and coping by gaming, and lower problem-focused and emotion-focused coping. Dysfunctional coping and coping by gaming were associated with perceived stress and depression in both IGD and control groups. Problem-focused coping was negatively associated with perceived stress and depression in controls. Conclusion: Individuals with IGD had higher perceived stress and depression, as they were more likely to cope with stress by dysfunctional coping and gaming and less likely to try problem-focused coping, particularly those with lower resilience. Interventions for IGD should promote problem-focused coping, such as active coping and planning strategies, particularly among those with lower resilience.

## 1. Introduction

Internet gaming is one of the most popular leisure activities globally [1]. However, some susceptible people lose control over gaming as a leisure activity, resulting in negative consequences, such as Internet gaming disorder (IGD). Approximately 0.21–57.50% of people have IGD [2]. The Diagnostic and Statistical Manual of Mental Disorders Fifth Edition (DSM-5) first included IGD as a research criterion [3]. The International Classification of Diseases 11th Revision (ICD-11) included gaming disorder (GD) as a formal diagnosis considering its effect on public mental health [4]. Maladaptive coping with stress in the form of gaming behavior can contribute to IGD [5,6,7]. Thus, the motivation for gaming escapism is the most significant motivational predictor of GD with clinical utility [8,9]. However, stress, coping, escapism, resilience, and depression have complex interactions in the development of IGD. Further research to explore these interactions can help develop practical intervention for individuals with IGD.

### 1.1. Coping by Gaming and Coping Strategy of IGD

Addictive behaviors are often initiated as maladaptive mechanisms for coping with stress [10]. Escape motivation and coping by gaming are associated with IGD severity symptoms [11,12]. King et al. [13] suggested that gaming motivation is a key factor for IGD development. However, the direct association between coping by gaming and IGD has not been confirmed by conducting a diagnostic interview study. Furthermore, whether coping by gaming benefits or negatively affects individuals with IGD remains unclear.

Individuals with IGD have been reported to experience higher stress [7,14]. A coping strategy mediates the association between stress and depression or anxiety [15,16], the two common comorbid psychiatric symptoms of IGD [17,18]. Thus, gamers use gaming as a coping strategy to escape from stress, implying that coping with stress is a critical issue that needs to be addressed in the treatment of IGD [7]. Evaluation of the coping strategy characteristics of IGD might provide information regarding the type of coping strategy that should be promoted for individuals of IGD to prevent their escaping behavior.

### 1.2. Resilience and Perceived Stress of IGD

Stress is a risk factor for the development of addiction, and serves as a cue that triggers relapses [19]. However, not all individuals have a maladaptive response to stress. Resilience has been defined as a personal trait to cope with adversity and protect one from its negative impact to achieve good adjustment [20]. Thus, resilience is assumed to buffer the effect of stress on the risk of addiction, and intervention for addiction should include resilience promotion [21]. Studies have demonstrated that perceived stress is positively correlated with IGD severity and negatively correlated with resilience [14,22]. An interview study revealed that individuals with IGD had higher perceived stress and lower resilience [7]. On the basis of these studies, Canale et al. [14] recommended enhancing online gamers’ psychological resilience to reduce their likelihood of experiencing higher stress levels as a result of gaming for many hours per week. However, the association between resilience and coping strategy of individuals with IGD has not been well-evaluated. An analysis of this association may provide insights into the type of coping strategy that should be promoted for IGD individuals with low resilience.

### 1.3. The Role of Resilience and Coping Strategy in Stress or Depression of IGD

Vulnerability to stress, such as having low resilience or using a dysfunctional coping strategy, contributes to depression [15,16]. Although a study demonstrated that individuals with IGD have low resilience [7], it remains unknown whether resilience or coping strategy is associated with depression among them. Moreover, how individuals with lower resilience cope with stress remains poorly understood. Understanding how resilience or coping strategy relates to depression or IGD severity may provide this information [23].

### 1.4. The Hypothesis

In this study, we hypothesized that (1) individuals with IGD have higher stress and depression; (2) they were more likely to cope with gaming or a dysfunctional strategy rather than a problem-focused strategy under stress condition; (3) individuals with IGD had lower resilience, and the resilience could buffer the stress or depression among those with IGD; and (4) coping with gaming and dysfunctional gaming were associated with depression and perceived stress among them. Thus, we evaluated coping by gaming, characteristics of the coping strategy, resilience, perceived stress, and depression among individuals with IGD and controls to test the aforementioned hypotheses.

## 2. Methods

### 2.1. Participants

Individuals with IGD (the IGD group), matched regular gamers (RGs), and non-gamers were recruited through advertisements around university campuses and on the online bulletin board systems of universities in Taiwan. In the IGD group, we included individuals who were aged between 20 and 38 years, had >12 years of education, played online video games for ≥4 h per day on weekdays and ≥6 h per day on weekends, and maintained a consistent pattern of Internet gaming for >2 years. Individuals who met these inclusion criteria participated in a diagnostic interview based on IGD criteria defined in the DSM-5 to confirm their diagnosis.

RGs and non-gamers were frequency matched by sex and age (±3 years) with participants in the IGD group. Participants who had a nonessential Internet use of <4 h per day [24], with this duration not involving regular gaming, were included as non-gamers. Participants who participated in regular online gaming (≥3 days a week) without fulfilling the diagnostic criteria of IGD were included as RGs.

The diagnostic interview comprised three parts: (1) diagnostic interviews for IGD based on the DSM-5 criteria; (2) the Chinese version of the Mini-International Neuropsychiatric Interview (C-MINI) [25] to exclude participants with psychotic disorders, bipolar I disorder, and substance use disorders; and (3) a history-taking interview to exclude participants with mental retardation, severe physical disorder, and brain injury. Sixty-nine participants were included in the IGD group and 138 in the control group (69 RGs and 69 non-gamers) after obtaining their informed consent. This study was approved by the Institutional Review Board of Kaohsiung Medical University Hospital, Taiwan (KMUHIRB-SV(II)-20150081).

### 2.2. Measures

DSM-5 diagnostic criteria for IGD [3]: The DSM-5 criteria comprise nine items: preoccupation, withdrawal, tolerance, unsuccessful attempts to control, loss of other interests, continued excessive use despite psychosocial problems, deception regarding online gaming, escape, and functional impairment [3]. We developed a semi-structured interview to examine the severity and frequency of each DSM-5 criterion for IGD. Participants fulfilling ≥5 criteria were included in the IGD group [26].

C-MINI: We conducted a diagnostic interview based on the modules of psychotic disorders, bipolar I disorder, and substance use disorders by using the C-MINI to determine their existence.

Brief Coping Orientations to Problems Experienced scale: Carver designed the Brief Coping Orientations to Problems Experienced (COPE) scale to assess various coping styles [27]. The scale consists of 28 questions, including 14 subscales in a Likert scale format (0 to 4 points). These subscales included active coping, instrumental support, planning, acceptance, emotional support, humor, positive reframing, religion, behavioral disengagement, denial, self-distraction, self-blame, substance use, and venting. Each subscale contains two questions, and represents a conceptually different coping style. On the basis of previous studies, we divided the whole scale into three dimensions. The problem-focused coping dimension included active coping, planning, and instrumental support. The emotion-focused coping dimension included emotional support, religion, positive reframing, acceptance, and humor. The dysfunctional coping dimension included self-blame, venting, denial, self-distraction, behavioral disengagement, and substance use [28,29,30].

Coping by gaming questionnaire (CGQ): The item for substance use was revised to evaluate coping by gaming as “I’ve been playing games to make myself feel better,” “I’ve been playing games to help me get through it,” and “I’ve been playing games to think about it less” (Appendix A).

Fourteen-item resilience scale: The 14-item resilience scale, which can present reliable internal consistency and external validity, was developed to evaluate the level of resilience, a positive personality characteristic strengthening personal adaptation, in the general population. Participants’ resilience was assessed using a 14-item resilience scale [31]. According to the authors, scores are calculated using a summation of response values for each item, thus enabling scores to range from 14 to 98. Scores <65 indicate low resilience; 65–81, moderate resilience; and >81, high resilience [31]. In the present study, participants scoring <65 were included in the low resilience group, and those scoring ≥65 were included in the adequate resilience group.

Perceived Stress Scale: The Perceived Stress Scale (PSS) was designed to measure the degree to which situations in one’s life are perceived as stressful. PSS scores are correlated with life event scores, depression, and physical symptomatology [32]. The PSS has adequate reliability to be an outcome measure of experienced levels of stress. In this study, the PSS-10 was used to evaluate the level of stress experienced by participants.

Center for Epidemiological Studies’ Depression Scale: The 20-item Mandarin Chinese version [33] of the Center for Epidemiological Studies’ Depression Scale (CES-D) [34] is a self-administered evaluation assessing participants’ frequency of depressive symptoms over the past week. Cronbach’s alpha of CES-D in the present study was 0.78. In this study, it was utilized to evaluate depression.

Process: All participants completed the diagnostic interviews and questionnaire assessment.

### 2.3. Statistical Analysis

The chi-squared test was used to evaluate the association among gender, low resilience, and IGD. An independent *t* test was conducted to evaluate differences in age and scores of the Brief-COPE scale and its subscales, the CGQ, PSS-10, and CES-D, with a Holm–Bonferroni correction. Logistic regression analysis was used to evaluate the association between resilience, coping styles, and IGD after controlling for gender and age. Furthermore, linear regression analysis was used to evaluate the association between coping styles and coping by gaming among IGD groups or controls. An independent *t* test was used to compare the scores of the Brief-COPE scale, CGQ, PSS-10, and CES-D between low and adequate resilience groups. Pearson’s correlation was used to evaluate the associations between the scores of these scales; *p* < 0.05 was considered significant. All statistical analyses were performed using SPSS version 20.0 (IBM Corp., Armonk, NY, USA).

## 3. Results

A total of 69 participants were classified into the IGD group. Women comprised 21.7% of each group (Table 1). No differences were observed in age between these two groups (Table 2).

### 3.1. Coping Strategies of the IGD Group

The independent *t* test evaluated the difference in score of subscales of the brief COPE between the IGD group and controls in Table 2. The results revealed that the IGD group had higher scores in denial, substance use, behavior disengagement, venting, self-blame, and gaming items, and lower scores in active coping, planning, and acceptance items than did the control group. Logistic regression analysis regressed IGD on the score of subscales of the brief COPE after controlling for gender and age in Table 3. The results revealed that the most associated coping items were venting (OR = 2.48; 95% CI = 1.78–3.45), active coping (OR = 0.36; 95% CI = 0.22–0.57), self-blame (OR = 1.51; 95% CI = 1.11–2.06), and behavior disengagement (OR = 1.52; 95% CI = 1.10–2.09) (Table 3). The difference in three dimensions of the brief COPE between the IGD group and controls was evaluated by the independent *t* test. The IGD group had higher dysfunctional coping and lower problem-focused coping than did the control group, but no difference was noted in emotion-focused coping. Logistic regression analysis evaluated the association between dimensions of the brief COPE and IGD. It revealed that dysfunctional coping was the most associated dimension of coping (OR = 1.34; 95% CI = 1.22–1.48), followed by problem-focused coping (OR = 0.7; 95% CI = 0.59–0.82). Thus, individuals with IGD were more likely to cope with stress by using dysfunctional strategies and less likely to cope by using problem-focused strategies.

### 3.2. Coping by Gaming in the IGD Group

The independent *t* test evaluated the difference in score of the CGQ between the IGD group and controls in Table 2. The results demonstrated that participants with IGD had higher scores in the CGQ. The results indicated that they were more likely to use gaming to cope with stress than controls. Linear regression regressed the score of the CGQ on the score of subscales of the Brief-COPE with control of age and gender (Table 4). The results revealed that a dysfunctional coping style was associated with coping by gaming in both the control and IGD groups. It indicated that those with a higher dysfunctional coping style were more likely to cope with stress by gaming. In the IGD group, participants with higher scores in venting and self-distracting had higher scores in the CGQ.

### 3.3. Resilience in the IGD Group

The chi-squared analysis evaluated the association between IGD and low resilience in Table 1. It demonstrated that a higher proportion of individuals in the IGD group (47.8%) had lower resilience than those in the control group (20.3%). Logistic regression analysis to regress IGD on low resilience revealed that individuals with lower resilience had 3.58 times (95% CI; 1.90–6.72) the odds of having IGD (Table 3). It implied a strong association between low resilience and IGD.

The independent *t* test evaluated the difference in scores of subscales of the Brief-COPE, CESD, and PSS between individuals with low resilience and others among IGD group in Table 5. The low resilience group had lower scores in active coping, humor, and acceptance (Table 4) than the adequate resilience group. They also had lower problem-focused and emotion-focused coping than the adequate resilience group, but no significant difference was noted in dysfunctional coping. Furthermore, they had higher perceived stress and depression than did the adequate resilience group.

### 3.4. Association among Coping by Gaming, Coping Style, Perceived Stress, and Depression in the IGD Group

Pearson’s correlation evaluated the associations between the scores of dimensions of the Brief-COPE, CGQ, PSS-10, and CES-D in both the IGD and control group in Table 6. The dysfunctional coping style and coping by gaming were positively associated with perceived stress and depression, where problem-focused coping was negatively associated with perceived stress (Table 6). Problem-focused coping was negatively associated with depression only in the control group.

## 4. Discussion

### 4.1. Coping Strategy of Individuals with IGD

The severity of Internet addiction is positively associated with negative or avoidance coping, and negatively associated with problem-focused coping [35]. The present study revealed the characteristics of the coping strategy of individuals with IGD. In particular, individuals with IGD were more likely to cope with stress by using dysfunctional coping (especially disengagement, venting, and self-blame), and less likely to cope by using problem-focused coping; they were least likely to use active coping.

Our findings demonstrated that individuals with IGD had higher perceived stress. Individuals with IGD have been reported to experience negative consequences, such as academic failure, social interaction difficulty, or job problems, under uncontrolled gaming [26]. Their dysfunctional coping styles, such as disengagement, may lead them to experience more challenges in their work and academic activities. They may blame themselves for dysphoria as a result of these problems, and frequently vent to their friends or in online forums. The unresolved stress and persistent dysphoria may result in a vicious cycle. This is compounded by the finding that they are unlikely to use active coping strategies to resolve their problems. Our results indicated higher perceived stress and depression in individuals with IGD, and the correlation between depression and dysfunctional coping can support this vicious cycle in individuals with IGD.

### 4.2. Coping with Stress by Gaming

Many studies have demonstrated that the motivation to use gaming for coping [11] and escaping [6,12] is associated with IGD severity. In line with these studies, our data revealed that individuals with IGD had higher scores in coping by gaming. To prevent the group effect in the analysis, we demonstrated the correlation between dysfunctional coping strategies and coping by gaming in both the control and IGD groups. This result suggests that people who cope with stress by using dysfunctional strategies are more likely to cope with stress by gaming. Further analyses in the IGD and control groups demonstrated that coping by gaming was associated with higher perceived stress and depression. Since gaming provides a space to satisfy the gamer with achievement and social interaction under a rewarding design, individuals under stress could keep gaming to escape from the frustration of the real world. However, those more likely to escape to gaming experience higher stress and depression. We could not conclude that gaming is a dysfunctional coping mechanism, because it could be secondary to stress and depression. However, our results did not support that gaming could effectively relieve the stress. On the other hand, problem-focused coping was negatively associated with perceived stress and depression in the same analysis for controls. Thus, the correlation between coping by gaming and stress and depression might indicate a bidirectional association between them. Furthermore, venting and self-distraction were correlated with coping by gaming among individuals with IGD. Thus, gaming may be used to cope with stress by venting emotions, such as anxiety and anger, distracting oneself from stress. However, these approaches are not effective in improving their mood or resolving the problems secondary to excessive gaming. The association between coping by gaming and dysfunctional coping strategies might partly explain the association between coping by gaming and depression.

### 4.3. Resilience of IGD

Low resilience is associated with IGD [7,14,22,36]. Yen et al. [7] demonstrated that individuals with IGD and low resilience had more severe depression. Our data support the association between low resilience and IGD. Two studies have established that mood status and depression mediate the association between IGD and resilience [7,36]. A questionnaire study indicated that perceived stress correlated with gaming hours only among those with low resilience [14], and an interview study did not find the buffer effect of resilience on the association between stress and IGD [22]. Because stress and depression may be not only the precipitating factors of IGD, but also the consequences of IGD, it is difficult to test the buffer effect of resilience on stress in IGD. Our finding that individuals with adequate resilience had lower stress and depression may support its buffering effect on stress and depression in individuals with IGD.

In the present study, individuals with IGD and with lower resilience had lower problem-focused and emotion-focused coping strategies than those with adequate resilience. Furthermore, IGD individuals with adequate resilience had higher problem-focused coping than did those with lower resilience (Table 4); they were more likely to use active coping, humor, and acceptance to cope with stressful situations under excessive gaming. Thus, although they also experience the negative consequences of IGD, they are more likely to stabilize their mood. This might explain why they had lower perceived stress and lower depression than those with low resilience among the IGD group. Our data thus support that resilience can exert a buffer effect on the depression and perceived stress of individuals with IGD through the adoption of problem- and emotion-focused coping mechanisms. On the other side, 52% (36/69) of individuals with IGD had adequate resilience. This suggested that other factors, such as high impulsivity, or environmental factors might be considered to contribute to the risk of IGD.

### 4.4. Limitations

First, the diagnosis of IGD was reached by relying solely on participants’ responses in a psychiatric interview. Additional information gathered from other sources, such as parents or partners, may contribute more information. Second, the cross-sectional analysis precluded the determination of a causal association between coping style, stress, and depression. Third, the sample size was limited, particularly for females, because of the difficulty in recruiting the case group.

## 5. Conclusions

Our results demonstrated that individuals with IGD experience higher stress and depression, probably because they use a dysfunctional coping style and cope with stress by gaming. It supports the role of gaming escapism in the emotional difficulty of IGD. They were less likely to adopt problem-focused coping strategies, such as active coping and planning, and these should thus be promoted among them. The role of gaming in coping with stress, such as venting or self-distracting, should be discussed in the intervention to promote other effective coping strategies. Furthermore, individuals with IGD having low resilience experience higher stress and depression, as they were less likely to use problem-focused and emotion-focused coping mechanisms. Thus, the intervention for coping with stress should be designed based on their resilience status, and more efforts should be made to encourage those with lower resilience to adopt healthier coping strategies.

## Figures and Tables

**Table 1:** The chi-squared analysis for the associations between gender, low resilience, and internet gaming disorder (IGD).

Variables	IGD Diagnosis		χ2 Test
	Yes (*n* = 69) *n* (%)	No (*n* = 138) *n* (%)	
**Gender**			
Female (*n* = 45)	15 (33.3)	30 (66.7)	0.000
Male (*n* = 162)	54 (33.3)	108 (78.3)	
Low Resilience ^a^			
Yes (*n* = 61)	33 (54.1)	28 (45.9)	16.78 ***
No (*n* = 146)	36 (24.7)	110 (75.3)	

*** <0.001; ^a^ Low resilience: scoring <65 in the 14-item resilience scale.

**Table 2:** The independent *t* test for the differences in age, coping strategy, perceived stress, and depression between the internet gaming disorder (IGD) group and control group.

Variables	IGD Group (*n* = 69)	Controls (*n* = 138)	*t* Test
	Mean ± SD	Mean ± SD	*t* Value
Age	25.73 ± 3.78	25.32 ± 4.20	0.24
Self-distraction ^a^	6.26 ± 1.00	6.22 ± 1.01	0.24
Active coping ^a^	5.64 ± 1.04	6.35 ± 0.93	−4.96 ***†
Denial ^a^	3.94 ± 1.50	3.34 ± 1.21	3.11 **†
Substance use ^a^	3.41 ± 1.81	2.67 ± 1.15	3.06 **†
Emotional support ^a^	5.83 ± 1.41	5.82 ± 1.35	0.03
Instrument support ^a^	5.94 ± 1.33	6.18 ± 1.26	−1.26
Behavior disengagement ^a^	4.86 ± 1.41	3.84 ± 1.10	5.70 ***†
Venting ^a^	6.19 ± 1.20	4.87 ± 1.29	7.07 ***†
Positive reframing ^a^	4.65 ± 1.17	4.36 ± 0.79	1.85
Planning ^a^	5.99 ± 1.28	6.70 ± 0.95	−4.51 ***†
Humor ^a^	5.58 ± 1.51	6.02 ± 1.30	−2.19 *
Acceptance ^a^	6.00 ± 1.10	6.58 ± 0.93	−3.98 ***†
Self-blame ^a^	5.83 ± 1.47	5.01 ± 1.20	4.25 ***†
Religion ^a^	3.09 ± 1.38	3.47 ± 1.63	−1.68
Coping by Gaming ^b^	10.17 ± 1.61	5.46 ± 2.65	15.83 ***†
Problem focus ^c^	17.57 ± 2.61	19.22 ± 2.34	−4.63 ***†
Emotion focus ^c^	25.14 ± 4.28	26.25 ± 3.73	−1.92
Dysfunctional coping ^c^	30.48 ± 4.45	25.96 ± 3.74	7.67 ***†
Perceived stress ^d^	22.46 ± 6.14	16.10 ± 5.55	7.50 ***†
Depression ^e^	23.91 ± 8.62	12.58 ± 7.98	9.38 ***†

* < 0.05, ** <0.01; *** <0.001; † Significant in the Holm–Bonferroni method; ^a^ the score of subscales of the Brief Coping Orientations to Problems Experienced scale (COPE); ^b^ the score of the coping by gaming questionnaire; ^c^ the score of dimensions of the Brief-COPE; ^d^ the score of the Perceived Stress Scale-10; ^e^ the score of the Center for Epidemiological Studies Depression Scale.

**Table 3:** Results of logistic regression analysis for the association between resilience or coping strategy and internet gaming disorder (IGD).

Variables	Wald	Exp (β)	95% CI
Model 1: resilience			
Age	0.20	0.98	0.91–1.06
Gender	0.06	1.10	0.52–2.30
Low Resilience	15.67 ***	3.58	1.90–6.72
Model 2: subscale of COPE			
Age	0.33	1.03	0.94–1.13
Gender	0.76	1.53	0.59–3.99
Venting ^a^	28.95 ***	2.48	1.78–3.45
Active coping ^a^	18.84 ***	0.36	0.22–0.57
Self-blame ^a^	6.78 **	1.51	1.11–2.06
Behavior disengagement ^a^	6.51 *	1.52	1.10–2.09
Model 3: dimensions of COPE			
Age	0.30	0.98	0.89–1.07
Gender	0.47	1.35	0.57–3.16
Dysfunctional coping ^a^	35.62 ***	1.34	1.22–1.48
Problem focus ^a^	17.70 ***	0.70	0.59–0.82

* <0.05; ** <0.01; *** <0.001; ^a^ The score of the subscales of Brief Coping Orientations to Problems Experienced (COPE) scale.

**Table 4:** Results of linear regression analysis to regress coping by gaming ^a^ on coping strategies among the internet gaming disorder (IGD) group or the control group.

Variables	Standardized Beta	*t*	*p*
IGD group			
Age	0.12	1.07	0.28
Gender	−0.06	−0.51	0.62
Dysfunctional coping ^a^	0.40	3.49	0.001
IGD group			
Age	0.11	1.12	0.27
Gender	−0.05	−0.47	0.64
Venting ^b^	0.45	4.32	<0.001
Self-distraction ^b^	0.28	2.67	0.01
Control group			
Age	−0.20	−3.06	0.003
Gender	0.07	0.80	0.42
Dysfunctional coping ^b^	0.50	6.95	<0.001

^a^ The score of the coping by gaming questionnaire. ^b^ The score of the subscales of the Brief Coping Orientations to Problems Experienced scale.

**Table 5:** Differences in coping strategy, perceived stress, and depression between individuals with low resilience and others in the internet gaming disorder group.

Variables	Low Resilience *n* = 33 Mean ± SD	Adequate Resilience *n* = 36 Mean ± SD	*t* Value
Self-distraction ^a^	6.12 ± 0.96	6.39 ± 1.05	−1.10
Active coping ^a^	5.12 ± 0.99	6.11 ± 0.85	−4.45 ***†
Denial ^a^	4.09 ± 1.51	3.80 ± 1.51	0.79
Substance use ^a^	3.15 ± 1.58	3.64 ± 1.99	−1.12
Emotional support ^a^	5.67 ± 1.34	5.97 ± 1.48	−0.90
Instrument support ^a^	5.76 ± 1.09	6.11 ± 1.51	−1.11
Behavior disengagement ^a^	5.00 ± 1.32	4.72 ± 1.49	0.82
Venting item ^a^	5.97 ± 1.05	6.39 ± 1.32	−1.47
Positive reframing ^a^	4.36 ± 1.08	4.92 ± 1.20	−2.00
Planning ^a^	5.55 ± 1.20	6.39 ± 1.23	−2.88 **
Humor ^a^	4.97 ± 1.49	6.14 ± 1.31	−3.47 **†
Acceptance ^a^	5.45 ± 1.00	6.50 ± 0.94	−4.47 ***†
Self-blame ^a^	6.27 ± 1.33	5.42 ± 1.50	2.50 *
Religion ^a^	2.88 ± 1.19	3.28 ± 1.52	−1.20
Coping by Gaming ^b^	8.39 ± 2.93	6.47 ± 3.20	4.05 ***†
Problem focus ^c^	16.42 ± 2.03	18.61 ± 2.66	−3.81 ***†
Emotion focus ^c^	23.33 ± 3.80	26.81 ± 4.06	−3.66 ***†
Dysfunctional coping ^c^	30.61 ± 4.22	30.36 ± 4.71	0.23
Perceived stress ^d^	25.36 ± 5.03	19.81 ± 5.91	4.19 ***†
Depression ^e^	27.61 ± 8.12	20.53 ± 7.70	3.72 ***†

* <0.05; ** <0.01; *** <0.001; † Significant in the Holm–Bonferroni method; ^a^ the score of subscales of the Brief Coping Orientations to Problems Experienced scale (COPE); ^b^ the score of the coping by gaming questionnaire; ^c^ the score of dimensions of the Brief-COPE; ^d^ the score of the Perceived Stress Scale-10; ^e^ the score of the Center for Epidemiological Studies Depression Scale.

**Table 6:** Correlation between coping strategy, perceived stress, and depression among the internet gaming disorder (IGD) group and controls.

Variables	Perceived Stress ^a^	Depression ^b^
Among IGD group		
Problem focus ^c^	−0.24 *	−0.13
Emotion focus ^c^	−0.18	−0.03
Dysfunctional coping ^c^	0.31 **	0.46 **
Coping by gaming	0.26 *	0.28 *
Among control group		
Problem focus ^c^	−0.28 **	−0.32 **
Emotion focus ^c^	−0.14	−0.15
Dysfunctional coping ^c^	0.42 **	0.36 **
Coping by gaming	0.25 **	0.30 **

* <0.05; ** <0.01; ^a^ The score of the Perceived Stress Scale-10; ^b^ The score of the Center for Epidemiological Studies Depression Scale; ^c^ The score of subscales of the Brief Coping Orientations to Problems Experienced scale.

## Data Availability

The data are not publicly available due to privacy.

## Appendix A. Coping by Gaming Questionnaire

These items deal with what extent you’ve been gaming to deal with stress or problems in your life. Make your answers as true FOR YOU as you can.

1 = I haven’t been doing this at all

2 = I’ve been doing this a little bit

3 = I’ve been doing this a medium amount

4 = I’ve been doing this a lot

1. I’ve been playing games to make myself feel better.
2. I’ve been playing gaming to help me get through the difficult times.
3. I’ve been playing gaming to think about the problems less.
